# Radiation safety considerations for the use of radium-224-calciumcarbonate-microparticles in patients with peritoneal metastasis

**DOI:** 10.3389/fmed.2023.1058914

**Published:** 2023-02-08

**Authors:** Simen Rykkje Grønningsæter, Johan Blakkisrud, Silje Selboe, Mona-Elisabeth Revheim, Øyvind Sverre Bruland, Tina Bjørnlund Bønsdorff, Stein Gunnar Larsen, Caroline Stokke

**Affiliations:** ^1^Division of Radiology and Nuclear Medicine, Oslo University Hospital, Oslo, Norway; ^2^Faculty of Medicine, University of Oslo, Oslo, Norway; ^3^Department of Oncology, Oslo University Hospital, Oslo, Norway; ^4^Institute of Clinical Medicine, University of Oslo, Oslo, Norway; ^5^Oncoinvent AS, Oslo, Norway; ^6^Department of Gastroenterological Surgery, Oslo University Hospital, Oslo, Norway; ^7^Department of Physics, University of Oslo, Oslo, Norway

**Keywords:** radium-224, radiation safety, alpha emitter, targeted alpha particle therapy, peritoneal metastasis

## Abstract

**Aim:**

Two ongoing phase I studies are investigating the use of radium-224 adsorbed to calcium carbonate micro particles (^224^Ra-CaCO_3_-MP) to treat peritoneal metastasis originating from colorectal or ovarian cancer. The aim of this work was to study the level of radiation exposure from the patients to workers at the hospital, carers and members of the public.

**Method:**

Six patients from the phase 1 trial in patients with colorectal cancer were included in this study. Two days after cytoreductive surgery, they were injected with 7 MBq of ^224^Ra-CaCO_3_-MP. At approximately 3, 24 and 120 h after injection, the patients underwent measurements with an ionization chamber and a scintillator-based iodide detector, and whole body gamma camera imaging. The patient was modelled as a planar source to calculate dose rate as a function of distance. Scenarios varying in duration and distance from the patient were created to estimate the potential effective doses from external exposure. Urine and blood samples were collected at approximately 3, 6, 24, 48 and 120 h after injection of ^224^Ra-CaCO_3_-MP, to estimate the activity concentration of ^224^Ra and ^212^Pb.

**Results:**

The patients’ median effective whole-body half-life of ^224^Ra-CaCO_3_-MP ranged from 2.6 to 3.5 days, with a mean value of 3.0 days. In the scenarios with exposure at the hospital (first 8 days), sporadic patient contact resulted in a range of 3.9–6.8 μSv per patient, and daily contact resulted in 4.3–31.3 μSv depending on the scenario. After discharge from the hospital, at day 8, the highest effective dose was received by those with close daily contact; 18.7–83.0 μSv. The highest activity concentrations of ^224^Ra and ^212^Pb in urine and blood were found within 6 h, with maximum values of 70 Bq/g for ^224^Ra and 628 Bq/g for ^212^Pb.

**Conclusion:**

The number of patients treated with ^224^Ra-CaCO_3_-MP that a single hospital worker - involved in extensive care - can receive per year, before effective doses of 6 mSv from external exposure is exceeded, is in the order of 200–400. Members of the public and family members are expected to receive well below 0.25 mSv, and therefore, no restrictions to reduce external exposure should be required.

## 1. Introduction

Peritoneal metastasis (PM) is most frequently caused by gastrointestinal and gynecological malignancies disseminating, and growing in serosa linings the abdominal cavity (1). The main treatment is cytoreductive surgery (CRS), often combined with hyperthermic intraperitoneal chemotherapy (HIPEC). Still there is a risk of recurrence of the disease.

Two ongoing phase I studies, RAD-18-001 (NCT03732768) and RAD-18-002 (NCT03732781), are investigating the use of radium-224 (^224^Ra) adsorbed in calcium carbonate microparticles (^224^Ra-CaCO_3_-MP) to treat peritoneal metastasis originating from colorectal and ovarian cancer. Patients at the highest planned activity level receive an injection of approx. 7 MBq of ^224^Ra intraperitoneally through a catheter, 2 days after CRS. Patients with PM with origin from colorectal cancer included in the RAD-18-002 trial also receive treatment with HIPEC after CRS.

**Figure 1:**
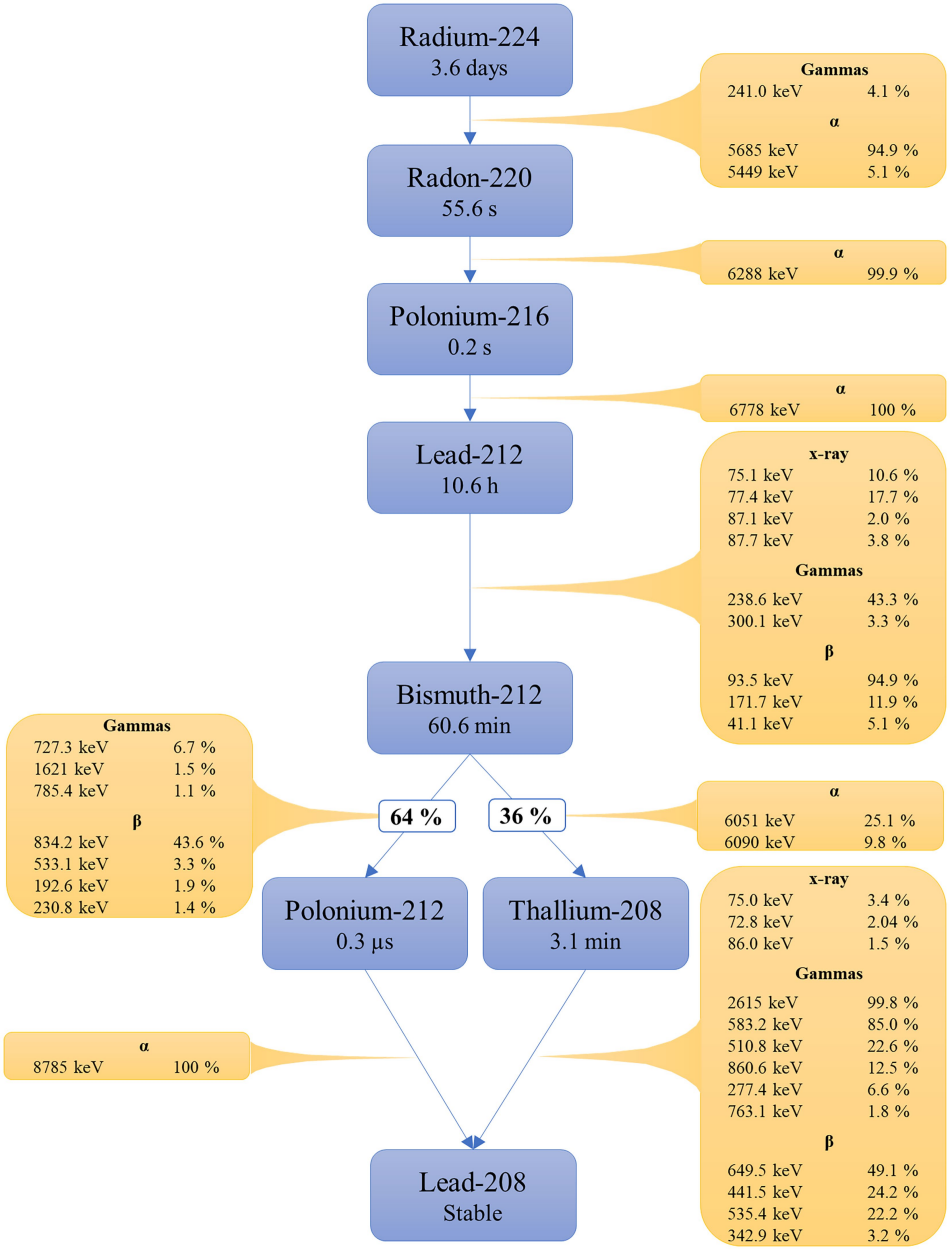
Overview of the ^224^Ra decay chain. Radiations with intensity <1% and photons with energy <70 keV are excluded. Data from the supplementary material of ICRP 107.

**Figure 2:**
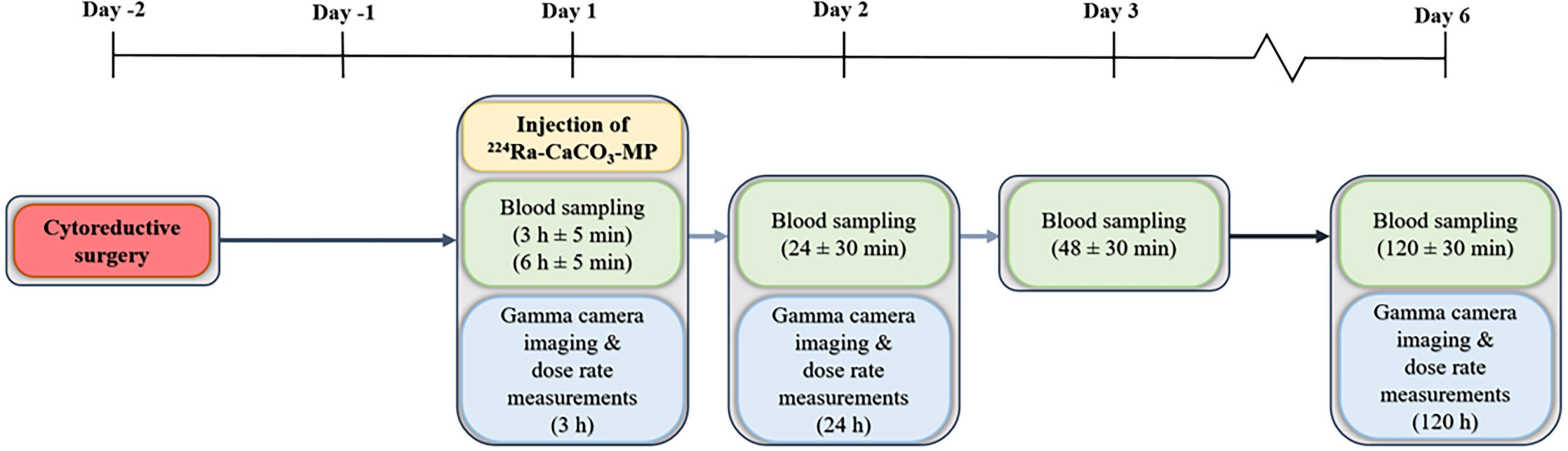
An overview of the treatment and measurement schemes for patients treated with ^224^Ra-CaCO_3_-MP.

The decay chain of ^224^Ra consists of radon-220 (^220^Rn), polonium-216 (^216^Po), lead-212 (^212^Pb), bismuth-212 (^212^Bi), polonium-212 (^212^Po, 64%), thallium-208 (^208^Tl, 36%) and stable lead-208 (^208^Pb; Figure 1; 2). ^212^Pb and ^208^Tl are the main photon emitters. ^212^Pb emits 77.4 keV x-ray photons (17.5%) and 239 keV γ-photons (43.6%), amongst others (2). The γ-photons of highest intensities with origin from ^208^Tl is 2,615 keV photons (99.8%) and 583 keV photons (85.0%). However, as ^212^Bi is branched, and 36% decays to ^208^Tl, the overall intensities of these photons are lower. ^224^Ra has a half-life (t_1/2_) of 3.6 days, while the daughters have shorter half-lives varying from

3·10^−7^ s to 10.6 h.

From the 1910’s, ^224^Ra has sporadically been used to treat ankylosing spondylitis (3), but to the best of the authors’ knowledge, no publications on the subject of radiation safety protection for ^224^Ra exist. Although not completely comparable, more research has been done in recent years on another isotope of radium; radium-223 (Ra^223^). Treatment fractions of 55 kBq/kg radium-223-dichloride ^223^RaCl_2_ is used for treating bone metastases with origin from metastatic castration resistant prostate cancer. Two publications have concluded that the product could be given on an outpatient basis, without restrictions on normal interactions and that patients do not need to follow specific restrictions related to radiation safety, as long as they attain to a set of hygienic precautions related to bodily fluids (4, 15).

For the treatment of peritoneal metastases, ^224^Ra is thought to be more suitable than ^223^Ra due to its shorter half-life, as it is expected that some of the injected radionuclide could be transported out of the peritoneal cavity (5). Hence, with a longer half-life more of the absorbed dose could potentially be deposited outside the peritoneal cavity.

The Council of the European Union sets in its Council Directive 2013/59/EURATOM effective dose limits for different categories of personnel, carers and the public (6). For example, the effective dose limit for occupational exposure is 20 mSv per year (the average over 5 years may be considered), while the limit for the public is 1 mSv per year.

The aim of the current study was to estimate radiation doses to hospital workers, carers and the public from patients receiving ^224^Ra-CaCO_3_-MP in a dosimetry cohort of six patients with peritoneal metastasis from colorectal cancer, undergoing measurements of external dose rates and radioactivity in urine and blood at several time points post treatment.

## 2. Methods

### 2.1. Patient population and ^224^Ra-CaCO_3_-MP treatment

Subjects with histologically confirmed colorectal carcinoma and peritoneal metastases eligible for CRS and HIPEC treatment were enrolled in a phase 1 trial to evaluate the dose, safety and tolerability of ^224^Ra-CaCO_3_-MP. For the current study, six patients from an expansion cohort at Oslo University Hospital were included. ^224^Ra was extracted from a generator consisting of thorium-228, which has a half-life of 1.9 years (7). ^224^Ra-CaCO_3_-MP was produced by Oncoinvent AS. A peritoneal catheter was inserted after surgery (day −2). ^224^Ra-CaCO_3_-MP, containing 0.7–1 g microparticles with nominally 7 MBq of ^224^Ra in equilibrium with daughters, was administered to the patients *via* the catheter at day 1 (Figure 2). Before injection, ^224^Ra-CaCO_3_-MP was diluted to 50 ml with Plasmalyte® (Baxter) isotonic solution. The suspension was injected intraperitoneally, and the injection was followed by a flushing with 200 ml of Plasmalyte®.

The study was approved by the Regional Committee for Medical and Health Research Ethics (REK). A written informed consent was given by all patients.

### 2.2. Dose rate measurements

Dose rate measurements were performed at day 1, 3 h after injection of ^224^Ra-CaCO_3_-MP, day 2 (24 h after injection) and day 6 (120 h after injection) with a hand held ionization chamber, a SmartION 2120S (Thermo Fisher Scientific, Waltham, Massachusetts, United States) with shielding cover. Dose rates were measured at a distance of 10 cm and 20 cm from the upper abdomen of the patients. Based on these measurements, mono-exponential curve fits were made in Matlab R2020b (MathWorks, Natic, Massachusetts, United States) to extra- and interpolate the dose rate of a patient as a function of time. Additionally, the curves were used to calculate the minimum, maximum and mean dose rates at each day.

To calculate the dose rate at other distances, the radiation source was assumed to be a flat finite plane with a radius *r* (8). A distance dependent ratio, *R*, for finding the dose rate at a distance *x_2_* is then given by


(Equation 1) $$R=\frac{\ln\left(\frac{r^{2}}{x_{2}{}^{2}}+1\right)}{\ln\left(\frac{r^{2}}{x_{1}{}^{2}}+1\right)}$$

### 2.3. Whole body measurements and effective half-life

Imaging was performed at day 1, day 2 and day 6 using a Siemens Symbia Intevo Bold gamma camera. A planar scan of 100 cm was acquired starting from the base of the skull, using medium energy collimators and a 240 keV energy window, and a 5% upper and lower scatter window. Automatic body contouring was used, and the scan had a 20 min acquisition time. Large regions of interest (ROIs) were drawn with margins around the patient on both the anterior and posterior images, using 3D Slicer version 4.8.1, revision 26,813 (The Slicer Community). The geometric mean of the counts found in the anterior and posterior ROIs were then calculated.

During the gamma camera imaging sessions, a measurement using a scintillator probe, RadEye SX with an FHZ 514A scintillation probe (Thermo Fisher Scientific, Waltham, Massachusetts, United States) was taken 50 cm from the upper abdomen, on the right side of the patient. Background measurements were also performed, and subtracted from the patient measurement to obtain the number of counts at each time point.

To estimate the whole body effective half-life of ^224^Ra-CaCO_3_-MP, three separate approaches were used. The gamma camera (the geometric means), SmartIon (the dose rates at 10 cm) and the RadEye (the number of counts). Separate mono-exponential curve fits and effective half-lives were calculated.

### 2.4. Scenarios

The mean, minimum and maximum dose rate measurements were used to evaluate radiation doses received by workers at the hospital, members of the family and the public from external exposure. Different scenarios were created, based on various assumptions regarding distance from and time spent with a patient. The day of discharge from the hospital varied for scenario 4.b, ranging from day 4 to day 12 (i.e., 6–14 days after surgery), with day 8 being the default for other scenarios.

1. Sporadic contact at the hospital: Assumes contact with patient on day 1, 2 and 6, with 10 min duration at 0.1 m and 15 min at 1 m. This could for instance reflect employees performing imaging or collecting patient samples, clinicians or nurses not involved in daily care, or employees involved in cleaning, transport of patients, etc. This scenario can also be relevant for family and friends visiting.
2. Daily contact at the hospital: Assumes daily contact with patient each day during day 1 to the day of discharge (day 8 as default). The subcategories a-c further divide the exposure according to the extent of daily contact. This may be relevant for employees at the hospital ward, involved in daily care, and possibly also visiting family members.
  - Low degree: 5 min at 0.1 m and 10 min at 0.5 m.
  - Moderate degree: 10 min at 0.1 m and 30 min at 0.5 m.
  - Extensive degree: 20 min at 0.1 m and 60 min at 0.5 m.
3. Sporadic contact after leaving the hospital: Separated in two scenarios, where the first assumes contact every third day, and the second one time encounters. The second may reflect prolonged transportation settings or similar occupations for members of the public.
  - Regular contact: 60 min of contact at 0.5 m every third day starting the day after discharge (day 9 as default).
  - Singular close contact: 3 h at 0.1 m at day 8.
4. Daily contact after leaving the hospital: Assumes frequent or prolonged contact starting at the day after discharge (day 9 as default). This may reflect family, or in some situations members of the public. The subcategories further divide the exposure according to the extent of contact.
  - Daily contact: 8 h at 1 m.
  - Close daily contact: 4 h at 0.1 m and 4 h at 1 m per day.

#### 2.4.1. Effective dose estimation equation

To estimate the effective dose, *H*, in these scenarios, the following equation has been used


(Equation 2) $$H=\underset{t_{d}}{\sum }{\dot{H}}_{t_{d}}\cdot \underset{x}{\sum }t_{x}\cdot R_{x}\;$$

where ${\dot{H}}_{t_{d}}$ is the dose rate of a given day (assumed constant each day for simplicity), *t_d_* is the number of days since the injection of ^224^Ra-CaCO_3_-MP included in the scenario, *t_x_* is the time (in hours) spent at a distance *x* from a patient, and *R_x_* is a distance dependent ratio for the distance x. The function $\dot{H}\left(t_{d}\right)$ is given by


(Equation 3) $${\dot{H}}_{t_{d}}={\dot{H}}_{0}\cdot e^{-\lambda_{e}t_{d}}\;$$

where ${\dot{H}}_{0}$ is the dose rate measured at 10 cm on day 1 and λ_e_ is the effective decay constant. ${\dot{H}}_{0}$ and λ_e_ varies for mean, minimum or maximum measurements. The distance dependent ratio, *R_x_,* is given by


(Equation 4) $$R_{x}=\frac{\ln\left(\frac{2025\;cm^{2}}{x^{2}}+1\right)}{3.056}\;$$

where *x* is the distance between the patient and the person in question. Equation 4 is found by using equation 1 and assuming a radius of 45 cm (*r^2^ = 2025 cm^2^*). The denominator of equation 1 thereby equals 3.056 for *x_1_* = 10 cm.

### 2.5. Fluid samples

Samples of fluids were collected approximately 3, 6, 24, 48 and 120 h after injection of ^224^Ra-CaCO_3_-MP. A minimum of 3 ml of urine and blood was collected, where the urine samples were collected from a urine collector bag. Each sample was measured at two different time points (time point 1 and 2) at least 48 h apart, with a Hidex Automatic Gamma Counter (Hidex, Turku, Finland), qualified for GxP analysis. The samples were weighted during analysis and consist of approximately 2.5 g for urine and blood, resulting in activity measurements given in Bq/g. The measurements at time point 1 were scheduled within 4 h of sampling from the patient, and time point 2 within 48–72 h after time point 1, when equilibrium between radium-224 and the progeny lead-212 has been established. The energy window was 60–110 keV with 10 min measurement time. Two measuring time points was used to estimate the amount of ^224^Ra.

The activity of ^224^Ra, $A_{Ra,t_{s}}$, in fluids was estimated using


(Equation 5) $$A_{Ra,t_{s}}=\frac{A_{Pb,t_{m2}}-A_{Pb,t_{s}}\cdot e^{-\lambda_{Pb}\left(t_{m2}-t_{s}\right)}}{\frac{\lambda_{Pb}}{\lambda_{Pb}-\lambda_{Ra}}\left(e^{-\lambda_{Ra}\left(t_{m2}-t_{s}\right)}-e^{-\lambda_{Pb}\left(t_{m2}-t_{s}\right)}\right)},$$

where $A_{Pb,t_{s}}$ is the activity of ^212^Pb at sampling,$\;A_{Pb,t_{m2}}$is the activity of ^212^Pb at time point 2, *(t_m2_-t_s_)* is the time between sampling and measurement number 2, and λ_Pb_ and λ_Ra_ are the decay constants of ^212^Pb and ^224^Ra, respectively. The decay constant is *λ = ln(2)/t_1/2_*, where *t_1/2_* is the physical half-life of the nuclide.

Bi-exponential curve fits were created for the measurements of ^224^Ra and ^212^Pb in blood and urine. Using the wash-out phase of the curves, the effective half-lives of ^224^Ra and ^212^Pb in urine and blood for this phase was calculated for each patient.

### 2.6. Hand exposure

Radiation doses to the hands of hospital workers receiving, preparing and injecting 7 MBq of ^224^Ra was measured at two occasions early in the study, using ring thermos-luminescence dosimeters (TLDs). Additionally, electronic personal dosimeters placed 5 cm from the glass containing ^224^Ra during vortexing, were used to record doses on three occasions.

## 3. Results

### 3.1. Patient group and protocol deviations

Of the six included patients, the median age was 61 years, and the average weight and height was 74 kg and 165 cm (Table 1).

**Table 1:** Characteristics of the patients included in the study.

Patient number	Gender	Age	Weight [kg]	Height [cm]	Injected ^224^Ra [MBq]
21-017	Female	68	65	158	7.23
21-020	Female	56	92	177	6.99
21-021	Male	66	82	178	7.07
21-023	Female	43	59	164	7.23
21-024	Female	28	67	164	7.38
21-025	Female	68	80	150	7.08

The 6 h blood measurement was not attainable for patient 21-017, and the 48 h gamma camera scan was not collected for patient 21-024. The procedures scheduled for day 2 and 6 was for patient 21-021 collected at day 3 and 7. However, the collection of fluid samples at 24 h after injection, was performed as planned.

### 3.2. Effective half-life

**Figure 3:**
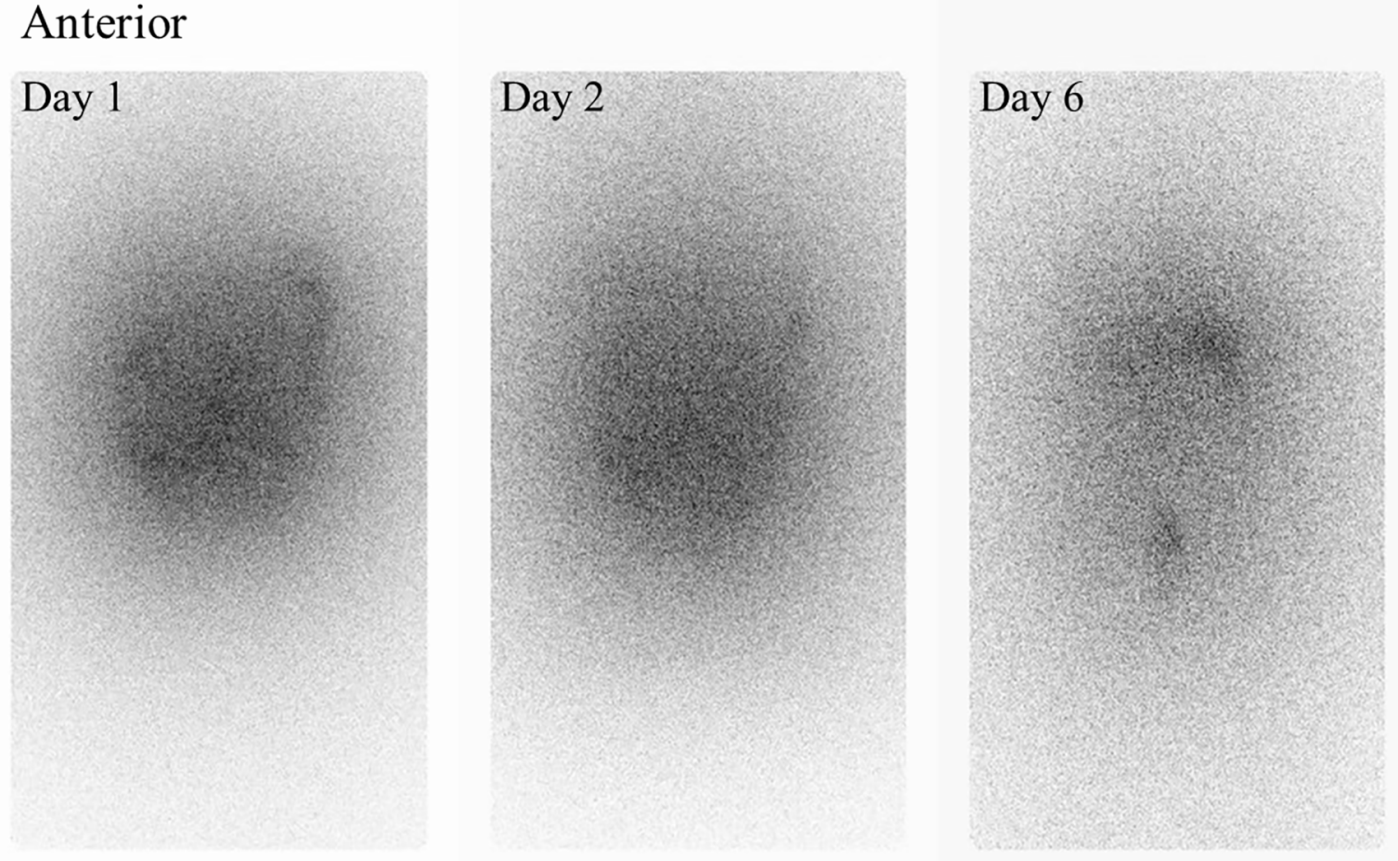
Anterior whole body images collected at day 1, 2 and 6.

The effective half-life estimated from whole body planar acquisitions (WB), scintillator probe (RadEye) and ionisation chamber (SmartION) measurements are shown in Table 2. There was overall a fair agreement between the three measurement techniques. The effective half-life for the whole body planar acquisitions, the RadEye and SmartION measurements was estimated to 3.2 d, 3.0 d and 2.8 d, respectively. Anterior whole body measurements acquired at day 1, 2 and 6 are shown in Figure 3.

**Table 2:** Effective half-life for the six patients included in the study, based on measurements with whole body gamma camera (WB), scintillator counter (RadEye) and ionization chamber (SmartION).

	t_effective_ [d]		
Patient number	WB	Radeye	Smartion
21-017	2.8	3.1	3.2
21-020	3.8	2.8	3.2
21-021	3.6	3.1	2.3
21-023	3.4	4.2	3.5
21-024	2.6	2.2	2.4
21-025	3.2	2.6	2.4

### 3.3. Dose rate measurements

**Figure 4:**
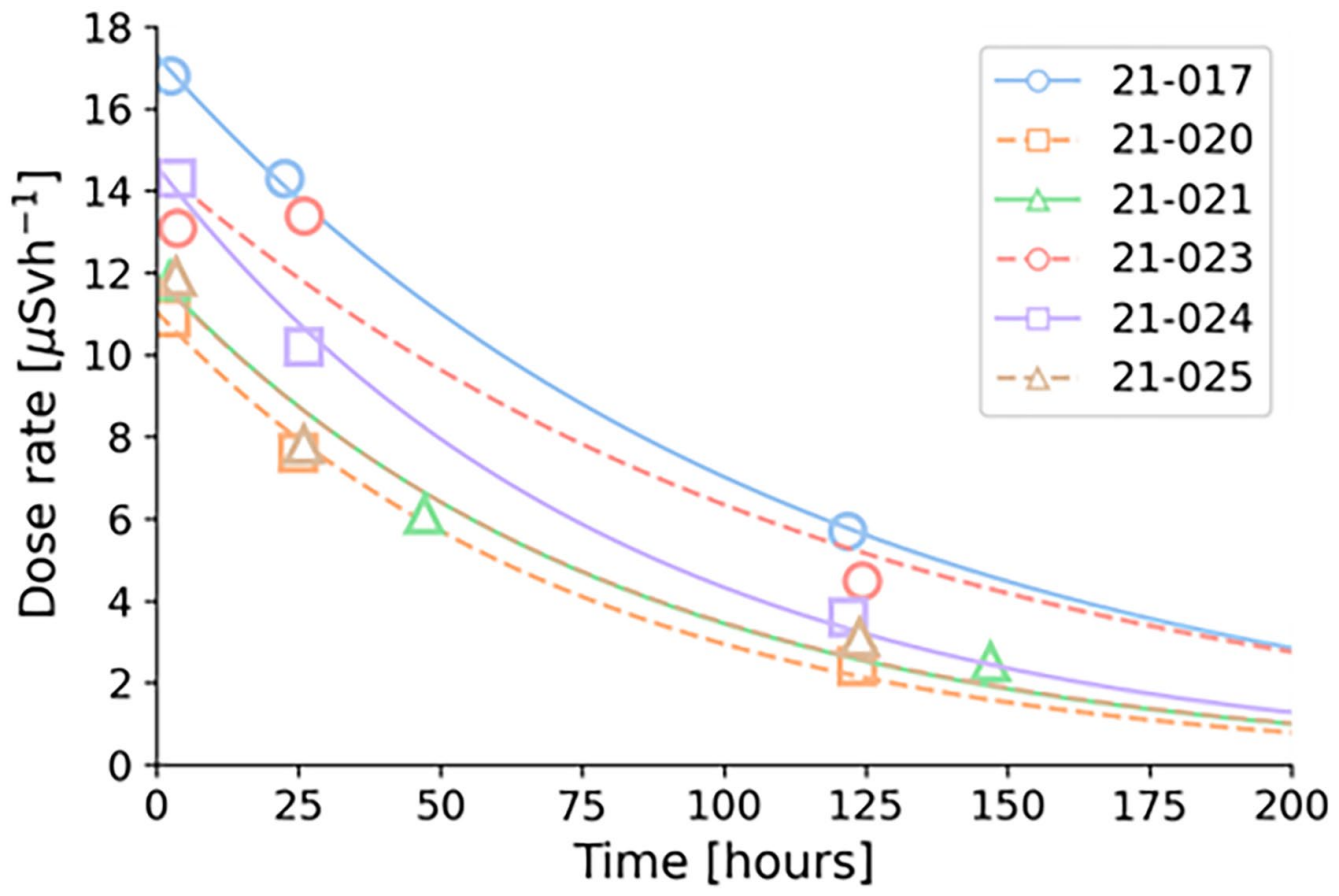
Dose rate measurements at 10 cm with corresponding curve fits. Each patient is indicated by a different colour, dots showing measurements and lines the fitted curves.

The dose rate measurements of the individual patients, and their fitted curves, are shown in Figure 4. The mean, minimum and maximum measurements at 3, 24 and 120 h after injection are summarized in Table 3.

**Table 3:** Mean, min, and max dose rate measurements at 10 and 20 cm for patients administered approx.

	10 cm			20 cm		
	Day 1	Day 2	Day 6	Day 1	Day 2	Day 6
Mean	13.1 uSv/h	10.4 uSv/h	3.7 uSv/h	7.9 uSv/h	6.4 uSv/h	2.6 uSv/h
Min	10.6 uSv/h	8.0 uSv/h	2.3 uSv/h	6.5 uSv/h	4.8 uSv/h	0.8 uSv/h
Max	16.8 uSv/h	13.9 uSv/h	5.9 uSv/h	9.8 uSv/h	8.2 uSv/h	3.7 uSv/h
CoV	18%	22%	41%	16%	20%	43%

7 MBq 224Ra. Based on the values of the fitted curves at 3, 24 and 120 h after injection, shown in Figure 2 for measurements at 10 cm.

#### 3.3.1. Estimated effective doses

The coefficients used in equation 2, for the estimations of effective dose, are shown in Table 4. The effective doses for different scenarios are shown in Table 5. While the patient was at the hospital, the highest effective doses were not surprisingly found for scenarios involving extensive daily contact, with an average effective dose of 22.7 μSv. The number of patients needed for a hospital worker to reach various effective doses were also calculated (Table 6), and, e.g., a moderate degree of daily contact would allow for approx. 350–700 patients per year before 6 mSv were reached. After leaving the hospital at day 8, close daily contact resulted in an average effective dose of 46.6 μSv, which would increase to 125 μSv if the patient left the hospital 4 days earlier, and decrease to 17.2 μSv if the patient left the hospital 4 days later. If someone would visit the hospital for all 4 days with extensive patient contact and have close daily contact after this, it would result in an average effective dose of 148 μSv. Transport, for 3 h at day 8 (scenario 3b), resulted in 7.3 μSv.

**Table 4:** Coefficients for use in equation 3 when estimating daily dose rate based on mean, min or max measurements.

	Mean	Min	Max
Ḣ_0_ [μSvh^−1^]	13.8	11.0	17.3
λ_e_ [d^−1^]	0.249	0.3172	0.2163

**Table 5:** Mean, min, and max effective doses received from one single patient for nine different scenarios.

	Effective dose (μSv/patient)		
Scenario	Mean	Min	Max
Sporadic contact at hospital (1)	5.0	3.9	6.8
Some daily contact at hospital (2.a)	6.1	4.3	8.4
Moderate degree of daily contact at hospital (2.b)	12.2	8.6	16.8
Extensive daily contact at hospital (2.c)	22.7	15.9	31.3
Sporadic contact after hospital, regular (3.a)	0.7	0.4	1.3
Sporadic contact after hospital, singular (3.b)	7.3	3.6	11.4
Daily contact after hospital (4.a)	8.6	4.0	14.6
Close daily contact; discharged at day 12 (4.b)	17.2	5.3	34.9
Close daily contact; discharged at day 8 (4.b)	46.6	18.7	83.0
Close daily contact; discharged at day 4 (4.b)	124.9	66.7	197.2

When not otherwise indicated, hospital discharge was assumed day 8.

**Table 6:** The number of patients that can be treated by a single individual before reaching 0.25, 1, 6 and 20 mSv, for the four different scenarios relevant for hospital workers.

	Number of patients before reaching limit							
	0.25 mSv		1 mSv		6 mSv		20 mSv	
Scenario	Min	Max	Min	Max	Min	Max	Min	Max
Sporadic contact at hospital (1)	65	37	259	148	1,551	886	5,170	2,952
Some daily contact at hospital (2.a)	58	30	234	119	1,403	714	4,675	2,379
Moderate degree of daily contact at hospital (2.b)	29	15	117	59	701	357	2,338	1,189
Extensive daily contact at hospital (2.c)	16	8	63	32	377	192	1,257	639

Patients are here assumed to leave the hospital at day 8.

### 3.4. Urine and blood samples

**Figure 5:**
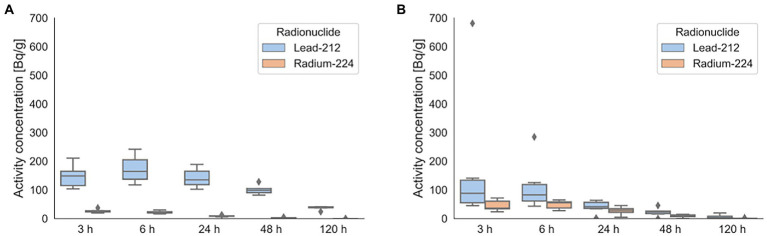
Activity concentration of ^224^Ra and ^212^Pb found in blood **(A)** and urine **(B)** for the six patients included. Dots indicate measurements that lies outside 1.5 of the interquartile range.

For urine, the highest measurements of ^224^Ra were commonly found at the first two time points, 3 h and 6 h, and ranged from approximately 30 to 70 Bq/g (Figure 5). For ^212^Pb, the peak values were usually found at the same time points, and ranged from 41 to 150 Bq/g, with one patient showing a higher value of over 600 Bq/g at 3 h. Urine samples showed an average (min-max) effective half-life during the wash-out phase of 0.7 d (0.3–1.3 d) for ^224^Ra. Due to high influx of ^212^Pb to urine, the effective half-life for the wash-out phase of the curve was 1.0 d in average (0.1–2.8 d).

Similarly for blood, the highest measurements of ^224^Ra was found at 3 to 6 h, and ranged from 22 to 40 Bq/g. For ^212^Pb in blood, the highest measurements was found at 6 h and ranged from 114 to 234 Bq/g. The effective half-life for the wash-out phase in blood was 0.6 d (0.4–0.8 d) for ^224^Ra and 2.1 d (1.3–2.9 d) for ^212^Pb.

### 3.5. Hand exposure

The doses from the TLDs were found to be lower than the limit of registration for the detectors (0.1 mSv). From the electronic personal dosimeters, doses of 1.3 μSv (0.41–2.05 μSv) were measured for vortexing and preparing the product.

## 4. Discussion

In this study, dose rate measurements of patients treated with ^224^Ra-CaCO_3_-MP gave an average dose rate of 13.1 μSvh^−1^ (10.6–16.8 μSvh^−1^) at 10 cm, approximately 3 h after injection. Since both alpha and beta particles will primarily stop in the tissue, this is mainly due to photons. Of the scenarios created, the highest effective dose, with a mean value of 46.6 μSv, were found for carers having close daily contact with a patient that was discharged at day 8 from the hospital, or a value of 124.9 μSv if the patient was released day 4. The number of patients treated with ^224^Ra-CaCO_3_-MP that a hospital worker - involved in extensive care - can receive per year, before effective doses of 1 mSv is exceeded, is in the order of 32–63, and is in the order of 639–1,257 before effective doses of 20 mSv is reached.

The Council of the European Union states, in its Council Directive 2013/59/EURATOM, an effective dose limit for occupational exposure in planned exposure situations of 20 mSv per year (6). This may for certain circumstances be extended to 50 mSv as long as the yearly exposure averaged over a period of 5 years do not exceed 20 mSv. The directive also states that personnel who are liable to receive more than 6 mSv per year should be individually monitored. For pregnant personnel, the radiation dose should not exceed 1 mSv from the pregnancy is discovered. The limit for the public is 1 mSv per year (6). Beyond this, there are also some national differences in dose constraints for hospital workers, carers, and members of the public. The European Council Directive requires that “Member States shall ensure that dose constraints are established for the exposure of carers and comforters, where appropriate.” For example in Norway, this has been implemented as that close family members may receive an effective dose of 1 mSv (children), 3 mSv (adults under 60 years of age), and 15 mSv (older adults) per treatment (9). In addition to Table 5 showing the effective doses received from exposure from a single patient, Table 6 shows the number of patients treated with ^224^Ra-CaCO_3_-MP that can be handled by employees in various scenarios, and different limits are hence included. Adaptations to other scenarios and limits can be done using equation 2 and Table 4.

The estimated effective dose for different scenarios allows an assessment for hospital workers, carers, and the public. The scenarios for sporadic and daily contact at the hospital were based on our experience during the trials. Only scenarios 1 and 2, involving sporadic and daily contact, are relevant for hospitals workers. As the overall clinical status and needs for support of individual patients vary, the category of daily contact was divided into three subgroups, ranging from some degree of contact (2a) to extensive degree of contact (2c). Highly conservative scenarios, including such as occupancy at 10 cm distance and prolonged contact, were also included as the patients may be in need of close clinical care. Table 6 show the number of patients that can be treated yearly in different scenarios before reaching dose limits of 0.25, 1, 6 and 20 mSv. It has been estimated that between 40 and 45 patients with colorectal cancer is a realistic number of patients treated yearly with ^224^Ra-CaCO_3_-MP at the Norwegian Radium Hospital. Hence, for most scenarios, the number of patients that can be treated before surpassing limits significantly outweighs the number of patients that are expected. Adding to this is that patient care of course is divided between several hospital workers, which will lower the individual exposure. However, with a limit of 1 mSv yearly, pregnant workers may theoretically surpass this limit if they were to single-handed treat more than 32 patients with need for extensive daily contact. Generally, in regard of the limits, it should be noted that all potential sources of exposure (numbers and types of other patients the employees are also treating) should be considered as a whole.

The patients undergo comprehensive surgery and can be expected to stay at the hospital for up to 2 weeks after surgery (day 12 after injection of ^224^Ra-CaCO_3_-MP). Day 12 was then investigated as a potentially time point of discharge, together with day 8 and day 4 Day 8 was here primarily used as the time of departure from the hospital based on our experience in the trial so far. Those with daily contact and close daily contact after discharge (scenario 4a and 4b) received effective doses up to 14.6 μSv and 83 μSv, respectively. If daily visits to the hospital are included (scenario 2c), they may receive up to 45.9 μSv and 110 μSv. If patients were to be discharged earlier, it would increase the effective dose to carers, and members of the public. In a setting where the patient leaves the hospital at day 4, instead of at day 8, the effective dose to those with close daily contact would increase to a maximum effective dose of 197 μSv, compared to 83.0 μSv for those discharged at day 8. This is still far below the dose limits, and do not generate any need for precautions.

In Norway, members of the public should not be exposed to more than 0.25 mSv from a single source of exposure (9). Members of the public may in contact with patients through several settings, and colleagues are typically often the most exposed. However, due to the strain the patients go through in relation to the treatment, they are not expected to go back to work for some time. If they were, the effective doses could be estimated through scenario 4a (daily contact) and would still be well below 0.25 mSv. Transportation, or other sporadic settings with contact, will only result in negligible contributions. E.g. after 3 h contact at 10 cm (scenario 3b), a member of the public will receive <4.6% of their yearly limit of 0.25 mSv. Even a continuous exposure at 20 cm for 4 weeks after the patient leaves the hospital at day 8, which is not a realistic scenario even for family members, would only result in up to 0.28 mSv and it is therefore no need for any restrictions for patients regarding the public.

Different approaches can be used to estimate the dose rate as a function of distance. A common method, the inverse square law, assumes a point source, which is not realistic for patients with activity distributed throughout the peritoneal cavity, as seen in Figure 3. Hence, it was assumed that a finite plane source would be more appropriate in the case of ^224^Ra-CaCO_3_-MP. The distance dependent ratio, *R*, was then given by equation 1. Although a source with a diameter of 90 cm will not truly represent the typical patient size, the agreement between the model and the measurements at 20 cm were very good, we therefore chose this diameter to avoid an underestimation of the dose rate for larger distances. Ideally, measurements at increased distances should have been included as well, to validate the model further. However, in this study, it was challenging to measure dose rates at more then 10 and 20 cm from the patients, since dose rates at larger distances would approach the background level.

Except for the use of ^223^RaCl_2_ to treat bone metastasis with origin from castration resistant prostate cancer (10), radionuclide therapy using alpha emitters is still mostly in its research stage. A higher number of radiation safety studies have therefore been published for radionuclide therapies using beta-emitters or for diagnostic tracers. While other radionuclides have been studied for treating peritoneal metastasis with origin from ovarian cancer, such as ^90^Y-HMFG1 and ^211^At-MX35-F(ab’)_2_ being two of the candidates (11, 12), we have not been able to find radiation protection publications related to these treatments. Stefanoyiannis et al. (13) compared studies examining radiation exposure to caregivers from patients for different common radionuclide therapies. These included radiopharmaceuticals with iodine-131 (^131^I), yttrium-90 (^90^Y) and lutetium-177 (^177^Lu). The different studies varied in injected activity, number of patients, types of dosimeters used, disease treated and the duration of the study. For thyroid cancer (all studies used ^131^I), activities ranging from 1,004–11,100 MBq was given, but did not result in effective doses to caregivers higher than 1.1 mSv. For B-cell lymphoma and neuroblastoma, activities up to 23,310 MBq of ^131^I were used, up to 7,400 MBq of ^177^Lu was used, and up to 1,200 MBq of ^90^Y was used. This resulted in effective doses to caregivers of up to 3.81 mSv, with the mean effective dose being considerably lower. Although amounts of activity should not be directly compared in most circumstances, it is worth mentioning that in the case of ^224^Ra-CaCO_3_-MP, an activity of three or four orders of magnitude lower (7 MBq) is used. In summary, Stefanoyiannis et al. found that the doses was within the dose constraints of 5 mSv to home caregivers, recommended by the International Commission of Radiological Protection (ICRP) (13). They also highlighted the importance of giving specific instructions to caregivers, as the highest dose values were found when no instructions were given.

The standard activity dosage of ^223^RaCl_2_ is 55 MBq/kg body weight given intravenously in six administrations 4 weeks apart (14). In contrast, for ^224^Ra-CaCO_3_-MP, one administration of 7 MBq is given intraperitoneally. Dauer et al. published in 2014 a study on radiation safety considerations for ^223^RaCl_2_ (15). They reported a dose rate immediately after injection being 0.02 μSvh^−1^/MBq at 1 m distance from the patient, and concluded that ^223^RaCl_2_ could be given on an outpatient basis, without restrictions on normal interactions with friends, relations, or co-workers. The dose rates measured for ^224^Ra-CaCO_3_-MP was equivalent to 0.11 μSvh^−1^/MBq at 1 meter distance, at the day of administration, found by dividing the average measured dose rate by the injected activity. The difference between dose rates may be caused by highly different distribution between the radiotherapeuticals (primarily blood pool versus only peritoneal cavity), resulting in a higher amount of radioactivity closer to the dose rate meter for ^224^Ra-CaCO_3_-MP, and the different radiations emitted. Furthermore, the kinetics of ^224^Ra-CaCO_3_-MP and ^223^RaCl_2_ may change the dose rates differently over time. While the majority of ^224^Ra-CaCO_3_-MP is expected trapped in the peritoneal cavity, some is transferred into the blood stream (Figure 5A). The two isotopes of radium is expected to chemically behave the same way and should follow the same biodistribution pathways after entering the blood stream (16). Differences lie in nuclear properties, like radiation energies and the physical half-life; which is 11.4 days for ^223^Ra and 3.6 days for ^224^Ra. Dauer et al. (15) found that for ^223^RaCl_2_ up to 60% of the injected activity was bound in the skeleton within 4 h after injection. While this is probably somewhat less than the percentage of ^224^Ra-CaCO_3_-MP remaining in the peritoneal cavity, the main factor contributing to different dose rates over time is most likely the different physical half-life of the two isotopes.

Findings reported by Serencsits et al. (4) support those of Dauer for ^223^RaCl_2_, but also stresses the importance of proper equipment for radiation protection and detection, as well as training of hospital workers to avoid contamination. They also conclude that patients do not need to follow specific restrictions related to radiation safety, as long as they attain to a set of hygienic precautions related to bodily fluids. An example of hygienic precautions would for instance be to flush twice after using the toilet. For both isotopes of radium, urine and fecal excretion are the two main excretion routes. Studies of ^223^RaCl_2_ showed that the cumulative excretion of urine was about 2% 48 h after injection, while the cumulative fecal excretion was 13% (1–25%) after 48 h and 64% (29–95%) after 72 h (17, 18). Dauer et al. (15) suggested that personnel involved in surgery, up to 2 m after injection of ^223^RaCl_2_, to take no extra precautions other than to be aware to reduce contamination. For ^224^Ra-CaCO_3_-MP, measurements of ^224^Ra and ^212^Pb in urine (Figure 5) show amounts below 40 Bq/g of ^224^Ra in blood and 90 Bq/g in urine. Higher activity concentrations are found for ^212^Pb, with up to 250 Bq/g found in blood and 140 Bq/g in urine (measurement for one patient up to 700 Bq/g). Still, while these are low amounts, both patients, carers and hospital workers potentially involved in handling fluids should be informed and instructed in best practice.

Radon gas may potentially be emitted from the patient through exhalation or from excreted fluids (16). Yamamoto et al. (19) found in a study investigating the detection of alpha emitting daughters of ^223^Ra, that the increase of alpha emitters in air were lower than the daily variation and therefore not an important source of radiation exposure. However, the gaseous daughter of ^223^Ra, ^219^Rn, has a half-life of 3.96 s, while ^220^Rn have half-life of 55.6 s. This may lead to a higher exposure from ^220^Rn. Since the amount ^224^Ra activity in urine was here found below the limit of what is considered radioactive (10 Bq/g) (6) already after 48 h, release from urine is most likely a minor issue. Exposure to radon gas is also relevant if re-surgery of the peritoneal cavity is required. While this has not been measured in this study, previous investigations of ^224^Ra in liquid volumes indicate that the mean diffusion length of ^220^Rn is limited to 300–400 μm, and hence only a small amount of ^220^Rn will have the potential to evaporate (20).

Radiation dose from photon contributions to hands is not considered an issue for ^224^Ra-CaCO_3_-MP, as the yearly dose limit to hands is set to 500 mSv (21). However, one should follow standard precautions for handling alpha-emitters to avoid contamination of the skin.

In summary, due to the low dose rates from the patients and low amount of activity found in blood and urine, no precautions related to external exposure should be required for patients treated with ^224^Ra-CaCO_3_-MP. The number of patients hospital workers can treat before exceeding an effective dose of for instance 6 mSv is 200–400 for patients with the need for extensive care. This is considered a worst-case scenario and significantly outweighs realistic number of patients.

## Data availability statement

The raw data supporting the conclusions of this article will be made available by the authors, without undue reservation.

## Ethics statement

The studies involving human participants were reviewed and approved by Regional Committee for Medical and Health Research Ethics (REK). The patients/participants provided their written informed consent to participate in this study.

## Author contributions

SG and CS conceived and developed most of the experiments. SG and SS performed or supervised the measurements by probes and gamma camera. TB planned and supervised the bioanalysis. SL was responsible for the patient inclusion and treatment. M-ER for administration of the radiotherapeutical. CS, M-ER, TB, and ØB supervised the project. SG and JB performed the data analysis and calculations. SG, JB, and CS wrote the first draft of the manuscript, including tables and figures. All authors contributed to the article and approved the submitted version.

## Funding

The clinical study is sponsored by Oncoinvent.

## Conflict of interest

ØB - Clinical consultant and shareholder - Oncoinvent AS. TB - Chief Scientific Officer and shareholder - Oncoinvent AS. JB has participated in a scientific advisory board of GE Healthcare.

The remaining authors declare that the research was conducted in the absence of any commercial or financial relationships that could be construed as a potential conflict of interest.

## Publisher’s note

All claims expressed in this article are solely those of the authors and do not necessarily represent those of their affiliated organizations, or those of the publisher, the editors and the reviewers. Any product that may be evaluated in this article, or claim that may be made by its manufacturer, is not guaranteed or endorsed by the publisher.

## References

[1] Coccolini F Gheza F Lotti M Virzi S Iusco D Ghermandi C. Peritoneal carcinomatosis. World J Gastroenterol. (2013) 19:6979–94. doi: 10.3748/wjg.v19.i41.6979, PMID: 24222942 PMC3819534

[2] ICRP. ICRP publication 107: nuclear decay data for dosimetric calculations. Ann ICRP. (2008) 38:7–96. doi: 10.1016/j.icrp.2008.10.004 19285593

[3] Priest ND Dauer LT Hoel DG. Administration of lower doses of radium-224 to ankylosing spondylitis patients results in no evidence of significant overall detriment. PLoS One. (2020) 15:e0232597. doi: 10.1371/journal.pone.0232597, PMID: 32353063 PMC7192484

[4] Serencsits B Chu BP Pandit-Taskar N McDevitt MR Dauer LT. Radiation safety considerations and clinical advantages of alpha-emitting therapy radionuclides. J Nucl Med Technol. (2022) 50:10–6. doi: 10.2967/jnmt.121.262294, PMID: 34750237 PMC9178548

[5] Westrom S Malenge M Jorstad IS Napoli E Bruland OS Bonsdorff TB. Ra-224 labeling of calcium carbonate microparticles for internal alpha-therapy: preparation, stability, and biodistribution in mice. J Labelled Comp Radiopharm. (2018) 61:472–86. doi: 10.1002/jlcr.3610, PMID: 29380410 PMC6001669

[6] Council Directive 2013/59/Euratom. Off J Eur Union. (2014) 57:13–14, 50–53. doi: 10.3000/19770677.L_2014.013.eng

[7] Li RG Lindland K Tonstad SK Bonsdorff TB Juzeniene A Westrom S. Improved formulation of (224) Ra-labeled calcium carbonate microparticles by surface layer encapsulation and addition of EDTMP. Pharmaceutics. (2021) 13:634. doi: 10.3390/pharmaceutics13050634, PMID: 33946852 PMC8145685

[8] Johnson HCT. Introduction to Health Physics. 4th ed New York, USA: The McGraw-Hill Companies, Inc. (2009).

[9] Norwegian Radiation and Nuclear Safety Authority, Østerås, Norway. Guidance for nuclear medicine. Guidance to “Regulations on Radiation Protection and Use of Radiation and regulations on radioactive waste and pollution”. Guidance no. 10. (2020).

[10] King AP Lin FI Escorcia FE. Why bother with alpha particles? Eur J Nucl Med Mol Imaging. (2021) 49:7–17. doi: 10.1007/s00259-021-05431-y, PMID: 34175980

[11] Hallqvist A Bergmark K Back T Andersson H Dahm-Kahler P Johansson M. Intraperitoneal alpha-emitting radioimmunotherapy with (211) at in relapsed ovarian cancer: long-term follow-up with individual absorbed dose estimations. J Nucl Med. (2019) 60:1073–9. doi: 10.2967/jnumed.118.220384, PMID: 30683761 PMC6681696

[12] Seidl C Essler M. Radioimmunotherapy for peritoneal cancers. Immunotherapy. (2013) 5:395–405. doi: 10.2217/imt.13.20 23557422

[13] Stefanoyiannis AP Ioannidou SP Round WH Carinou E Mavros MN Liotsou T. Radiation exposure to caregivers from patients undergoing common radionuclide therapies: a review. Radiat Prot Dosim. (2015) 167:542–51. doi: 10.1093/rpd/ncu338, PMID: 25431487

[14] Agency EM ed. Annex I-Summary of Product Characteristics. Amsterdam, Noord-Holland, Netherlands: European Medicines Agency; (2013).

[15] Dauer LT Williamson MJ Humm J O’Donoghue J Ghani R Awadallah R. Radiation safety considerations for the use of 223RaCl2 DE in men with castration-resistant prostate cancer. Health Phys. (2014) 106:494–504. doi: 10.1097/HP.0b013e3182a82b37, PMID: 24562070 PMC4981573

[16] ICRP. ICRP Publication 137: Occupational Intakes of Radionuclides: Part 3. Ottawa, Ontario, Canada: SAGE, International Comission on Radiological Protection; (2017).

[17] Yoshida K Kaneta T Takano S Sugiura M Kawano T Hino A. Pharmacokinetics of single dose radium-223 dichloride (BAY 88-8223) in Japanese patients with castration-resistant prostate cancer and bone metastases. Ann Nucl Med. (2016) 30:453–60. doi: 10.1007/s12149-016-1093-8, PMID: 27272279 PMC4961730

[18] Chittenden SJ Hindorf C Parker CC Lewington VJ Pratt BE Johnson B. A phase 1, open-label study of the biodistribution, pharmacokinetics, and dosimetry of 223Ra-dichloride in patients with hormone-refractory prostate cancer and skeletal metastases. J Nucl Med. (2015) 56:1304–9. doi: 10.2967/jnumed.115.157123, PMID: 26182965

[19] Yamamoto S Kato K Fujita N Yamashita M Nishimoto T Kameyama H. Detection of alpha radionuclides in air from patients during Ra-223 alpha radionuclide therapy. Sci Rep. (2018) 8:10976. doi: 10.1038/s41598-018-29449-9, PMID: 30030499 PMC6054680

[20] Napoli E Bonsdorff TB Jorstad IS Bruland OS Larsen RH Westrom S. Radon-220 diffusion from 224Ra-labeled calcium carbonate microparticles: some implications for radiotherapeutic use. PLoS One. (2021) 16:e0248133. doi: 10.1371/journal.pone.0248133, PMID: 33662039 PMC7932545

[21] ICRP. ICRP Publication 103: The 2007 Recommendations of the International Comission on Radiological Protection. Ottawa, Ontario, Canada: Elsevier, International Comission on Radiological Protection; (2007).

